# Editorial: Decision making and problem solving in organizations: assessing and expanding the Carnegie perspective

**DOI:** 10.3389/fpsyg.2024.1455794

**Published:** 2024-08-23

**Authors:** Pino G. Audia, Daniella Laureiro Martinez, Daniel A. Newark

**Affiliations:** ^1^Organizational Behavior, Tuck School of Business at Dartmouth College, Hanover, NH, United States; ^2^Management, Technology, and Economics, ETH Zurich, Zürich, Switzerland; ^3^Management & HR, HEC Paris, Jouy-en-Josas, France

**Keywords:** decision making, learning, goals, aspirations, decision premises, Carnegie perspective, organizations

## Introduction

Decisions steer the course of individuals, organizations, markets, and governments. It is therefore unsurprising that significant research efforts in economics, psychology, political science, and sociology are dedicated to understanding how they are made and how they can be improved. However, within this intellectual landscape, the large and growing body of work linked to what we refer to as the Carnegie perspective on decision making has increasingly been siloed (March, 1989, 1994; Argote and Greve, 2007; Posen et al., 2018). Despite the centrality of the Carnegie perspective to decision making and of decision making within the Carnegie perspective, the last edited collection on decision making to include Carnegie among its various perspectives dates back over 25 years (Shapira, 1997). Undoubtedly, the immense popularity of the heuristics and biases research program (Kahneman, 2011) has played a role in the diminished visibility of the Carnegie perspective. At the same time, the increased tendency to classify research within the Carnegie perspective as being about learning rather than decision making (Levinthal and March, 1993; Audia and Greve, 2021), when in reality both labels are likely apt, has also separated Carnegie from predominant scholarly conversations on decisions.

After these developments of recent decades, we think the time may be ripe for the Carnegie perspective to resume a more central place in decision making research in the social sciences for several theoretical reasons. First, the Carnegie perspective complements other theoretical perspectives by offering a more agentic take on decision making. For example, while sharing with the heuristics and biases program the premise that individuals are boundedly rational, it does not reduce decision making to an analysis of deviations from a narrow form of economic rationality stemming primarily from cognitive processes. Instead, it views individuals as problem solvers capable of adapting through the decisions they make to changes in their environments, though it identifies conditions that reduce this capacity. Second, by integrating learning from experience in the decision process, the Carnegie perspective may be well suited to assess the implications for decision making of the increased use of Artificial Intelligence, a technology that relies heavily on past actions and outcomes. Third, the Carnegie perspective tackles the central issue of attention allocation by proposing mechanisms that remain distinctive in comparison, for example, to the economic logic of the allocation of scarce resources. This confluence of issues presents a unique moment of reflection and openness to reconsidering and reshaping the academic dialogue on decision making. To that end, a key objective of this Research Topic is to facilitate an exchange between scholars within the Carnegie perspective on decision making and scholars studying decisions using other approaches.

## What is the Carnegie perspective on decision making? What is distinctive about it?

To define what we mean by the Carnegie perspective on decision making, we follow Scott and Davis (2015), who suggest that a theoretical perspective should be understood in two senses: (a) the intellectual foundations that created the context in which it developed and (b) the analytically enduring features that make it distinctive.

### Intellectual foundation

The intellectual foundation originated in the 1940s and 50s at Carnegie Tech (now Carnegie Mellon University) thanks to the seminal contributions of Herbert Simon, James March, and Richart Cyert. Three inter-related intellectual building blocks stand out in their seminal work: bounded rationality, behavioral realism, and multi-method orientation. First, Simon postulated (Simon, 1955, 1956, 1964, p. 2) “important constraints (in the decision process) arising from the limitations of the actor himself as an information processor” and referred to decision theories that incorporate such constraints as theories of bounded rationality. Like Cyert and March (1963) after him, he used stylized models of rational decision making that were dominant in economics as an alternative conception against which he articulated the notion of boundedly rational decision making. Second, from these initial steps came a commitment to behavioral realism, that is, to studying actual decisions and to developing models that reflect how people and organizations make actual decisions (Gavetti et al., 2007). As Cyert and March (1963, p. 2) put it: “the emphasis on studying actual decision processes implies a description of the firm's decision in terms of a specific series of steps used to reach that decision.” This commitment to behavioral realism brings to the fore steps in the decision process that are less prominent in other lines of decision research, such as the allocation of attention, the search for alternatives, and the resolution of conflict (e.g., conflict among goals and preferences). The result is a broader conception that integrates activities of problem solving (e.g., choosing issues that require attention) and decision making (e.g., choosing among alternative actions). Research in the Carnegie perspective generally asks, given limitations to individuals' cognitive abilities: (a) To which issues do they allocate their attention? (b) Once an issue becomes the focus of their attention and potentially a subject of a decision process, how do they identify and evaluate, through a process of search, alternatives among which to choose?, and (c) How are choices among endogenously generated alternatives made?

Third, tied to behavioral realism was also the orientation to combine a variety of methods to study decisions. This reflected the interdisciplinarity of Simon, March, and Cyert. Because their scholarly work and interests crossed the boundaries of psychology, sociology, organization theory, artificial intelligence, political science, and economics, they saw value in combining methods from different areas of the social sciences and balancing the concern for internal validity with the concern for realism. As a result, studies in this perspective are not as reliant on experiments involving students or participants in online research platforms as are other lines of work on decision making. Scholars within the Carnegie perspective complement experimental data (e.g., Laureiro-Martinez et al., 2015; Billinger et al., 2021) with analyses of archival data (e.g., Bromiley, 1991; Clough and Piezunka, 2020), qualitative research (e.g., Rerup, 2009), simulations (e.g., Gavetti and Levinthal, 2000; Baumann et al., 2019) and theoretical work (e.g., Newark, 2018). This multi-method orientation is unique within the decision research landscape. Overall, as numerous strands of decision research went on to build on the concept of bounded rationality, research in the Carnegie perspective pursued a distinctive path oriented by the combined influence of these intellectual building blocks.

### Enduring features

Considering core ideas laid out in the foundational work and the areas of study where greater progress has been made over the past two decades, we identify three analytically enduring features that make this perspective distinctive within the broader landscape of research on decision making. The first is the centrality of goals and aspirations (Cyert and March, 1963). Within this perspective, goals and aspirations influence several steps of the decision process: attention, conflict, search, and evaluations of alternatives. The starting point is that individuals generally seek to achieve multiple goals, and they resolve the potential conflict of doing so by focusing on goals sequentially, rather than simultaneously. Moreover, instead of choosing alternatives that maximize performance on a goal, individuals *satisfice* by choosing alternatives that exceed a desirable performance threshold known as the aspiration level. When performance on a goal is met, their attention moves to another goal for which performance is still below the aspiration level. Assessments of failure on such a goal triggers the search for a solution, which stops when a solution sufficient to meet the aspiration level is found.

Two recent developments have extended these ideas in important ways. The first is the recognition that low performance often is evaluated not in relation to an aspiration level but to a survival point, and shifts of attention between these two reference points have ramifications for choice (March and Shapira, 1992; Audia and Greve, 2006; Boyle and Shapira, 2012). The second is the integration of social psychological research on self-enhancement in the process by which performance is evaluated (Jordan and Audia, 2012), which has led to a more complete understanding of how multiple goals and alternative aspiration points influence decisions (Audia and Brion, 2007; Joseph and Gaba, 2015; Keil et al., 2023).

The second enduring feature is individual learning from experience, both own experience and others' experience (Levitt and March, 1988; Argote and Miron-Spektor, 2011). Decisions are not seen in isolation but within a flow of events that influences the definition of a problem, the formation of aspirations, the considered alternatives, and the desirability of each. As March (1988, p. 11) notes, “aspirations adapt to experience, generally rising with success and falling with failure—though not necessarily at the same rate. Aspirations also adapt to the experience of others within a reference group.” Carnegie scholars examine the implications of different updating rules of aspirations (Lant, 1992; Mezias et al., 2002) as well as conditions that guide the formation of different kinds of reference groups (Audia et al., 2022; Luger, 2023).

Experience also impacts the search for and the assessment of alternatives. The actions of comparable others add to the pool of alternatives considered when a decision is made (Greve, 1998; Baum and Dahlin, 2007). But a success trap can alter that process as individuals repeat decisions that have led to success in the past while failing to discover or recognize alternatives that would produce better outcomes (Levinthal and March, 1993; Audia et al., 2000). Conversely, “a hot stove” effect occurs when individuals immediately abandon alternatives that initially appear worse than they actually are (Denrell and March, 2001).

The third enduring feature is the idea of decision premises. As Simon (1947, p. 79) notes, “individual choice takes place in an environment of 'givens'—premises that are accepted by the subject as the basis for choice”. Routines are perhaps the most researched decision premises within the Carnegie perspective (Cyert and March, 1963; March and Olsen, 1989). They are patterns of behavior that arise in repetitive situations and enable individuals to simplify the decision process (Cohen and Bacdayan, 1994). Importantly, their use is not confined to the individuals who learned these patterns (March and Olsen, 1989). Organizations store routines often in the form of standard operating procedures which they transfer to individuals for whom routines become decision premises. Routines generally improve decisions, but they also hinder decisions when they guide choice in the wrong circumstances. To illustrate the negative effects of routines, Cohen and Bacdayan (1994), refer to situations in which room operators, airline pilots, and Soviet troops followed routines in the wrong situations. A recent wave of research moves beyond the influence of routines on decisions to examine the circumstances under which routines change (Feldman et al., 2016).

Directly building on the work of Simon and March, another important line of work on decision premises focuses on the influence of institutional logics—“a set of assumptions and values, usually implicit, about how to interpret reality, what constitutes appropriate behavior and how to succeed” (Ocasio, 1997, p. 196; Thornton and Ocasio, 1999; Thornton et al., 2012). Logics are conceived as a cultural mechanism that regulates the allocation of attention and the identification of desirable solutions. For example, studies show that logics influence risk behavior (Almandoz, 2014), the type of acquisitions firms make (Greve and Zhang, 2017), and the type of executives firms appoint (Thornton and Ocasio, 1999). In environments in which multiple logics coexist, socialization and work experience are often sufficient to capture variation in logics that have a measurable influence on the decision process.

### An example of what is distinctive about the Carnegie perspective

To provide an example of the ways in which the Carnegie perspective differs from other lines of work on decision making, consider the following decision scenario from Lovallo and Kahneman (2003): “In 1992, Oxford Health Plans started to build a complex new computer system for processing claims and payments. From the start, the project was hampered by unforeseen problems and delays. As the company fell further behind schedule and budget, it struggled, vainly, to stem an ever-rising flood of paperwork. When, on October 27, 1997, Oxford disclosed that its system and its accounts were in disarray, the company's stock price dropped 63%, destroying more than $3 billion in shareholder value in a single day.”

Lovallo and Kahneman use this and other examples to call attention to the influence on decisions of heuristics and biases such as the planning fallacy, anchoring, and competitor neglect (Kahneman, 2011; Thaler and Sunstein, 2021). While the cognitive processes they highlight undoubtedly play a part in guiding the decision process, the Carnegie perspective—through its focus on goals and aspirations, learning, and decision premises—offers a wider analytical lens. First, it asks whether this decision failure is a failure of attention. Decision makers might have overlooked problems and delays related to the implementation of the computer system because their attention might have been directed to other activities tied to other goals deemed more important. Alternatively, problems and delays in the implementation of the computer system might not have received attention because they might not have been evaluated to be below the goals and aspiration levels set for it. For example, some goals might have been met whereas others might have not. Likewise, some aspiration levels might have been achieved whereas others might have not.

Second, the Carnegie perspective asks whether this decision failure is a failure of learning. Here the focus is how own experience and others' experience influence the search for and the assessment of alternatives. Indeed, the Carnegie perspective holds that the process of generating alternatives does not occur in a vacuum. Past success in completing successful implementations potentially even when facing delays, for example, might have made this process myopic, stifling the generation of new alternatives and making some alternatives more desirable than others. Similarly, implementation challenges of the same computer system experienced by peer firms might have impacted the evaluation of lack of progress.

Third, the Carnegie perspective asks whether this decision failure is a decision premise failure. For example, if a standard operating procedure was in place to guide the implementation of a new computer system, the question is whether it included steps that would have prevented the decision failure. If such steps existed, this might have been an instance of routine decay. Carnegie research on routine change would then help identify conditions that might have led to this form of failure. Additionally, while it may not apply to this example, if multiple logics potentially coexisted in the decision environment under consideration, the Carnegie perspective also asks which institutional logic is likely influencing decision makers' allocation of attention and consideration of desirable solutions. Overall, as this example reveals, taking on a Carnegie perspective on decision making implies adopting an analytical lens that highlights the importance of the context and the endogenous nature of some of the key steps of decisions. Such a different understanding of the reasons behind this decision failure would lead to corrective actions that differ from those advanced by Lovallo and Kahneman.

## The papers in the Research Topic

The call for papers for the Research Topic was published in August 2022. We received a total of 39 submission, of which 11 were selected for publication. We accepted for publication the first paper in March 2023 and the last one in February 2024. This accelerated schedule, typical of Frontiers academic journals, was made possible by the contribution of many colleagues who agreed to complete reviews in an expedited fashion as well as the support of the staff of Frontiers in Psychology, particularly its Chief Editor, Prof. Treadway. Special thanks go to Giovanni Gavetti and Thorbjorn Knudsen for guest editing two papers submitted by members of the editorial team. The authors of the 11 papers are affiliated with institutions in Asia, Europe, Latin America, and North America, reflecting the international impact of the Carnegie perspective on decision making. Three papers are conceptual and eight are empirical. The latter illustrate the multi-method orientation of work done within the Carnegie perspective. They include experiments, quantitative field studies, meta-analysis of archival studies, lab-in-the-field methods, and simulations. The manuscripts draw on and extend central ideas within the Carnegie perspective on decision making.

The three conceptual pieces situate the Carnegie perspective on decision making in relation to other lines of work on decisions, identifying similarity and difference as well as opportunities for integration. In a conceptual piece, Levinthal and Newark locate the Carnegie perspective on decision making in relation to other lines of work in economics and psychology. They contrast different intellectual traditions along a key dimension, namely, how they characterize the context of decisions. In classical conceptions of rational choice in economics the context is external, taking the form, for example, of changes in relative prices. Context takes the form of framing in Kahneman and Tversky's heuristics and biases line of work and choice architecture in Thaler's work on nudges. Context is a decision makers' familiarity with their environment or choice in Gigerenzer's work on ecological rationality.

Using these distinct intellectual lenses as counterpoints, Levinthal and Newark suggest that Carnegie offers a characterization of context that is more social and more organizational. For example, learning from own and others' experience captures the social dimension of context, organizational structures and standard operating procedures are uniquely organizational, and recent work on institutional logics considers cultural influences. Yet, while emphasizing the influence of the context on decisions, the Carnegie perspective retains an important role for individual agency by recognizing that decision makers routinely face situations that require interpretation. Levinthal and Newark underscore the richness that accompanies Carnegie's fidelity to behavioral realism, as the Carnegie perspective attempts to incorporate the historical, social, structural, and interpretive dynamics of choice.

In another conceptual piece, Greve focuses on the influence of goals on decisions, one of the most productive lines of work and a key mechanism in the Carnegie perspective. He identifies three ways in which goals impinge on the decision process: regulating the allocation of attention through satisficing, enabling numeric control that triggers decisions, and guiding the search for alternatives. While noting that considerable evidence has accumulated on the influence of the profitability goal on a wide range of change decisions, he also points to evidence that, when goals are more specific, the change decisions tend to match the goals such as when low market share prompts change in market positions and accidents spur changes in safety. Besides confirming the influence of goals on the choice of alternative, the work on more specific goals documents the importance of multiple goals. While highlighting some progress, Greve sees greater gaps between theory and empirical evidence on the relationship between goals and search and how aspirations are formed.

To address these gaps and, more broadly, to advance Carnegie research on goals and decision making, he calls for more work on how decision makers integrate information from multiple goals and use it to direct search and make decisions. This echoes Levinthal and Newark who also view interpretation as a key process underlying individual agency in the Carnegie perspective. However, Greve uses the psychological term “construal” to emphasize the proximity between such development and contemporary research on social psychology. As an example of this construal research, he refers to work on self-enhancement that documents that multiple goals or aspirations open for multiple forms of construal can lead to inaction when performance is low enough to indicate a need for problemistic search.

Goals figure prominently also in Gaba and Joseph's conceptual piece on conflict. Like differences in perceptions of the internal and external environment, differences in goals are a key source of conflict that has the potential to interfere in the decision process, for example, by complicating the selection of action alternatives. Gaba and Joseph highlight progress on the identification of situations that create conflict stemming from these two sources. They point to active lines of work on multiple goals and cognitive representations as offering new insights on the implications of conflict for decision making. Importantly, unlike research in psychology that directly measures perceptions of conflict, in Carnegie, work conflict remains unobserved. This latent conception of conflict reflects the theoretical positions articulated by Cyert and March that conflict is an inherent characteristic of organizations that is never fully resolved. While Carnegie research, with its focus on context, offers an understanding of the situations that accentuate conflict and of the mechanisms by which conflict is reduced—sequential attention to goals, decentralization, slack, and coalition formation—Gaba and Joseph call for work that provides a more fine-grained and dynamic understanding of conflict, differentiating, for example, between latent conflict and expressed conflict. They suggest that integrating psychological research on the expression of conflict could offer an avenue for bringing in emotions in Carnegie research on conflict.

The eight empirical papers ground the broad conceptual picture and tackle specific propositions. In the spirit of the call for papers, each paper draws connections to relevant work outside Carnegie, particularly psychological research. Each paper also seeks to explore new angles on topics that have been and are still central within Carnegie. As our discussion of these papers show, there are fruitful connections between the papers that point to potential new themes.

Blettner et al. use meta-analysis methodologies to examine the relationship between performance and strategic decisions involving change, search, risk taking, and RandD investments. While this relationship has been studied using a variety of methods (e.g., experiments, case studies, and simulations), data availability and an interest in actual decisions made within organizations have steered a considerable volume of studies toward analysis of publicly listed firms. The assumption in this work is that the strategic decisions of publicly listed firms are largely the result of their Chief Executive Officers' deliberations. So, it should not surprise the reader that their meta-analysis includes 205 studies yielding 516 effect sizes and >3 million firm-year observations. Impressively, their baseline findings confirm the view that has recently been advanced in the literature that the influence of performance on decisions is more consistently seen when performance falls below the aspiration level. Indeed, in their data, changes in performance that is above the aspiration level are not significantly related to those strategic actions.

Among their key findings, Blettner et al. report that the relationship between low performance and strategic decisions is altered by two types of CEO knowledge—knowledge acquired through job experience and knowledge acquired through domain expertise. Job experience weakens the relationship between low performance and strategic actions, whereas domain expertise strengthens it. This difference arises, they argue, because, unlike domain expertise which gives access to a wide range of beliefs and actions, knowledge acquired through experience is more likely to be biased and to lead to overconfidence. They also consider two types of incentives—performance-based and compensation. Surprisingly, neither alter the relationship between strategic actions and low performance.

Carnegie research recognizes the influence on decisions of own experience and others' experience but understanding of how these two forms of experience combine is still relatively rare. Kim et al. tackle this topic by developing a simulation model that aims to account for irregular decision patterns in which entrepreneurs vacillate between self-employment and wage-employment. Their simulation shows that switches of attention from learning from own experience to learning from others' experience prompted by performance falling below the historical aspiration level may account for such patterns which they term “intermittent risk taking.” Instead of simply opting for the other available option when performance is poor, decision makers extend their assessment of alternatives by considering the outcomes of other decision makers. This may result in sticking to options that have not produced desirable results or in moving away from options that are meeting the aspiration level. Greve, in his conceptual piece, highlighted as an important research gap the question of how decision makers draw on own experience and others' experience to form aspiration levels and make decisions. Kim et al. study of irregular decision patterns in an entrepreneurial context is an example of the kind of advances that can be achieved by moving in that direction.

Liu and Tsay take a different look at learning from others' experience. Their focus is the extent to which decision makers can correctly infer useful knowledge from others' performance. The extreme case is a top performer whose positive outcomes are entirely due to luck. In that case, incorrectly inferring that the actions of the lucky top performer are advantageous would be detrimental. They pose the question: in a performance distribution, where is the greatest probability that performance does not match merit?

Previous studies suggest that the top performers are likely to be the luckiest, thus evidencing the greatest disconnect between performance and merit. Liu and Tsay undertake an analysis of performance and merit among academics to test their novel prediction that that gap between merit and performance is greatest in the middle of the performance distribution. Using publicly available citations to identify performance and a survey of academics to determine merit (i.e., impact), they find that the decoupling of performance and merit is greater for publications with moderately high success. They attribute this finding to early reinforcement processes. Early recognition due to authors' prestige or tight networks allow some papers to experience a reinforcement process that leads to many citations. However, the growth in citations is constrained by their lesser quality. Highest recognition requires both high quality and early recognition which suggests that some high-quality papers get stuck in the low end of the distribution. Within the broader Carnegie theme of the influence of vicarious learning on decision making, Liu and Tsay highlight the decoupling of performance from merit as a condition that may derail the decision process.

Koçak et al. seek to move Carnegie research toward a more direct consideration of conflict, in line with Gaba and Joseph's recommendations. Instead of assuming latent conflict and focusing on conditions that elevate it and the consequences of conflict, as much of previous Carnegie work has done, they conceptualize different types of cognitive conflict and examine their implications for coordination. Using the term “code” to denote fuzzy mappings between concepts adjusted through experience, they differentiate between causal codes—beliefs in what actions cause which outcomes—and moral codes—beliefs in what is evaluated as desirable. In their main experiment, they use two vignette scenarios, one concerning a proposal for opening a daycare facility and a second one concerning the evaluation of a carbon emission reducing technology and have online participants read the private and independent thoughts and opinions of two managers involved in the decision. Participants are then asked to complete a survey regarding relationship conflict, likelihood of reaching an agreement, likelihood of negative affect between the parties, and likelihood of negative engagement. Compared to no misalignment, both forms of misalignment negatively impact all conflict variables, but, in line with their prediction, moral code misalignment has a greater negative impact on all conflict variables than causal code misalignment. By linking cognitive representations, a growing literature within Carnegie, to conflict, this proof-of-concept study points to a new approach for expanding understanding of this central topic.

Stumpf-Wollersheim et al. examine the influence of emotions on the emergence of routines. Following Cohen and Bacdayan (1994), they focus on four dimensions of routines. The first three connote how a routine helps alleviate the constraints posed by bounded rationality: the degree of repetitiveness of action sequences; the speed with which those actions are taken; and the degree of reliability, defined as the extent to which the action sequences generate good outcomes. The fourth dimension captures the potential downside of routines, namely, the failure to change the action sequence when the need arises, which they term attentiveness in action. Stumpf-Wollersheim et al. focus on sadness and fear because these two emotions are often experienced during periods of change—fear of an unknown future and sadness for leaving a past state. Yet, they are different because sadness is associated with uncertainty acceptance whereas fear is associated with uncertainty avoidance.

They rely on an experimental design in which pairs play a computerized version of the Target the Two card game developed by Cohen and Bacdayan. They prime the emotions through recall of an experience and use music and pictures while participants play the game. They find that sadness increases repetitiveness, speed, and reliability but it reduces attentiveness in action. The effect of sadness on reliability was against their prediction. Also contrary to their predictions, fear does not influence repetitiveness, speed, and reliability, but, in line with their prediction, it increases attentiveness in action. They conclude that sadness fosters heuristic decision making, whereas fear fosters comparatively more-attentive team-level decision making.

Richner et al. study adaptive decision making in a lab-in-the-field-study involving officer cadets in the Swiss Armed Forces. Their interest lies in exploring the role of individual antecedents—personality traits and cognitive flexibility—and context-evoked antecedents—recent stress, present emotional states, and task motivation—on individuals' capacity to balance exploitation (i.e., learning and continuing to use solutions that have yielded good outcomes) and exploration (i.e., trying new solutions that may give superior results).

Carnegie research has shown that decision makers often fail to switch from exploitation to exploration particularly following success and when following standard operating procedures. Officer cadets are asked to complete a four-armed bandit task over 150 trials, which, analogous to war simulations, presents the challenge of balancing exploitation (i.e., learning and continuing to use slots with positive payout) and exploration (i.e., trying different slots that may yield better payouts). Exploration-exploitation performance equals total payout. Combining three of the indicators of cognitive flexibility in one factor—vigilance, working memory, and switching—Richner et al. find that cognitive flexibility is positively related to exploitation-exploration performance. Moreover, cognitive flexibility mediates the positive effects of emotional stability and context-evoked task motivation on exploration-exploitation performance. Emotional stability mediates the negative effect of context-evoked stress on cognitive flexibility.

Aggarwal et al. examine a construct similar to cognitive flexibility, cognitive versatility (i.e., the ability to shift in cognitive styles). Their focus is on fluid participation in teams—changes in team composition and skill sets. When people come and go, coordination failures that compromise decision making are more likely. A structural remedy to alleviate this bounded rationality gap is intersecting role sets in which some tasks are completed by more than one role. But, because organizations often lack people to fill intersecting roles, many teams are set up with disconnected role sets that complicate coordination and adaptation. Aggarwal et al. propose that the cognitive versatility of core members who have decision authority may compensate for the use of disconnected role sets. More cognitively versatile members attenuate the gap in performance between teams with disconnected role sets and teams with intersecting roles sets because they may rely on their cognitive versatility to make decisions that make the team more adaptive. Their analyses of a sample of 342 teams from a hospital Emergency Department support their proposition: teams with disconnected roles are less effective than teams with intersecting roles, as evidenced by longer hospital stays and more hand-offs for patients. But the presence of a cognitively versatile core member reduced the gap.

Guo et al. also focus on roles. Dating back to work by Guetzkow and Simon (1955) and March and Simon (1958), a central insight in the Carnegie perspective is that different types of structures alter communication in ways that influence coordination and decision making. But this view overlooks the possibility that the people occupying critical roles may not be a good match with the requirements of the position. In that scenario, structural arrangement alone may not be sufficient to deal with the constraints posed by bounded rationality. Guo et al. suggest that allowing group members to choose who occupies the central position in a centralized network creates a better match between the capacity of the chosen individual and the role. The reason is that, through working together, members learn who possesses which skills and can rely on this learning to identify the best match. They study 41 triads working on a complex programming task. All triads are allowed to express their choice but, in the position chosen condition, the triads were given their choice, whereas, in the position assigned condition, the triads were given a randomly selected person they did not choose. Communication activity both predicted selection to the central position and mediated the relationship between choice and team performance which was measured as the number of errors. This selection process, they conclude, complements the centralized network as a solution to the challenges posed by bounded rationality.

## New directions for the Carnegie perspective on decision making

We conclude by identifying new research directions that emerge from the papers in the Research Topic. The first research direction concerns the study of individual characteristics. As Greve (p. 6) provocatively said, research within Carnegie has been quite person-less. Indeed, most theorizing tends to be process oriented and focused on the influence of the context. However, as several papers in this Research Topic suggest, we are increasingly seeing a shift toward a more careful consideration of the person. The context remains prominent even within this new research direction. In fact, rather than examining the direct influence of individuals characteristics on decisions, an approach revealed by some of the studies in this Research Topic is to look at their influence within specific contexts, where individual differences may serve as a complement to structural arrangements that help cope with bounded rationality or as filters to contextual influences such as performance feedback. Studies on self-enhancement (Audia and Brion, 2007, 2023; Lauenstein et al., 2024), cognitive flexibility (Laureiro-Martinez and Brusoni, 2018), and job experience (Gaba et al., 2023) exemplify the emergence of this new research direction.

The second research direction is to integrate within the Carnegie perspective the study of emotions. Levinthal and Newark (p. 7) encourage Carnegie scholars to move away from the “Tin Man” sensibility of an actor without a heart by providing a better accounting of the role of emotions. Two papers in this Research Topic tackle this task. Richner et al. examine emotions as context-evoked (e.g., stress) and as individual traits (e.g., emotional stability) and find some evidence indicative of the importance of the latter in the decision process. Stumpf-Wollersheim et al. show how emotions such as sadness and fear may enter Carnegie theorizing through their influence on the emergence of routines and their negative effects. Together with earlier work on the influence of emotion of routine adoption (Dojback Hakonsson et al., 2016), these early efforts show the promise of pursuing this research direction.

A third research direction is the examination of interpretation in the face of ambiguity. Levinthal and Newark (p. 2) note how a focus on the influence of context implies recognizing the importance of interpretation when the signals coming from the context are not clear cut. Greve is even bolder, as he identifies construal as a central area for future work. In this Research Topic, an example of interpretation under ambiguity is the study by Kim et al., where learning from own experience and others' experience requires interpretation. In previous research, a notable example is the line of work examining the influence of multiple goals and multiple aspirations on decision making (Audia and Greve, 2021; Levinthal and Rerup, 2021). Levinthal and Newark also expand the view of interpretation as an antecedent of decision making to suggest that interpretation in the form, for example, of stories, allows individual to assign existential meaning to what they do (Newark, 2014). Although the empirical papers do not directly deal with these conceptions of interpretation under ambiguity, we view this as an important research direction.

Pursuing these three research directions will require integrating within the Carnegie perspective insights and findings generated by research in psychology and neuroscience. Whether examining cognitive versatility, sadness and fear, or conflict, the authors of the papers in this Research Topic undertook that task. The results are novel insights and findings that strengthen the explanatory power of the Carnegie perspective and simultaneously create opportunities for dialogue and increased visibility with other lines of work.
